# Personalized Alert Notifications and Evacuation Routes in Indoor Environments

**DOI:** 10.3390/s120607804

**Published:** 2012-06-08

**Authors:** Ignacio Aedo, Shuxin Yu, Paloma Díaz, Pablo Acuña, Teresa Onorati

**Affiliations:** Computer Science Department, Universidad Carlos III de Madrid, Avda. de la Universidad 30, 28911 Leganés, Madrid, Spain; E-Mails: 100286457@alumnos.uc3m.es (S.Y.); pdp@inf.uc3m.es (P.D.); pacuna@inf.uc3m.es (P.A.); tonorati@inf.uc3m.es (T.O.)

**Keywords:** alert notification systems, evacuation routes, mobile emergency systems

## Abstract

The preparedness phase is crucial in the emergency management process for reaching an adequate level of readiness to react to potential threats and hazards. During this phase, emergency plans are developed to establish, among other procedures, evacuation and emergency escape routes. Information and Communication Technologies (ICT) can support and improve these procedures providing appropriate, updated and accessible information to all people in the affected zone. Current emergency management and evacuation systems do not adapt information to the context and the profile of each person, so messages received in the emergency might be useless. In this paper, we propose a set of criteria that ICT-based systems could achieve in order to avoid this problem adapting emergency alerts and evacuation routes to different situations and people. Moreover, in order to prove the applicability of such criteria, we define a mechanism that can be used as a complement of traditional evacuation systems to provide personalized alerts and evacuation routes to all kinds of people during emergency situations in working places. This mechanism is composed by three main components: CAP-ONES for notifying emergency alerts, NERES for defining emergency plans and generating personalized evacuation routes, and iNeres as the interface to receive and visualize these routes on smartphones. The usability and understandability of proposed interface has been assessed through a user study performed in a fire simulation in an indoor environment. This evaluation demonstrated that users considered iNeres easy to understand, to learn and to use, and they also found very innovative the idea to use smartphones as a support for escaping instead of static signals on walls and doors.

## 1. Introduction

When an emergency occurs, emergency workers have to deal with an exceptional situation where activities and time to perform them are determinant for an effective response. The preparedness phase is crucial in the emergency management process for reaching an adequate level of readiness to react to potential threats and hazards [1]. During this phase, emergency plans are developed based on vulnerability analyses and response capability assessment in order to identify procedures and responsibilities to reduce effects of a disaster [1,2]. These plans depend on the kind of emergency, since procedures and needs vary according to the characteristics of the situation (*i.e.*, the circumstances are not the same for a fire in a building than for a tsunami).

According to US government Occupational Safety & Health Administration, an Emergency Action Plan (EAP) is a written document that facilitates and organizes employer and employee actions during workplace emergencies [3]. A good definition of the EAP makes possible to minimize the personal injuries and the damage of the infrastructure avoiding a chaotic response. Some of the mandatory components of an EAP are, among others, specific evacuation procedures, including routes and exits, and procedures for assisting visitors and employees to evacuate, particularly those with disabilities or who do not speak the local language. Moreover, alarm systems (like sirens or rotating lights) can be included in the EAP to notify employees (including disabled ones) to take different kind of actions, including evacuation.

Information and Communication Technologies (ICT) can improve the management of the evacuation procedures and the notification through alerts in working places by supporting the different characteristics and abilities of those involved in the emergency as well as the features of the emergency. As stated in [4], the technologies used for communicating and sharing data can influence the management of an emergency, improving the effectiveness and the efficiency of an Emergency Action Plan.

In order to guarantee an effective notification and evacuation for all people affected by an emergency, specific criteria related to user profiles, available technologies, kinds of emergency and contextual circumstances have to be identified. In this context, there are some proposals that try to achieve these criteria by using multimodal alerts (including HUENS [5], CUCEM [6] and cAlert [7]), but they do not take into account impaired individuals nor concepts such as understandability, accessibility and context awareness. Other interesting related works are the contributions from Koo *et al.* [8] and Manley *et al.* [9]. Their aim is to define simulation models to improve evacuation procedures considering individuals with disabilities though they are not focused on notifying and presenting information to end users. We propose a set of criteria for guaranteeing effective notifications and evacuation procedures based on the concepts of accessibility and usability. Moreover, for showing the applicability of such criteria we develop a mechanism that on the basis of a defined Emergency Action Plan provides personalized alert notifications and evacuation routes to users with smartphones in working places. This mechanism uses multimodal alerts to notify the emergency and provide evacuation routes to all kinds of users, including impaired people as well as contextually disabled who due to physical problems (such as noise or lack of visibility) or cognitive ones (inability to understand the language in which the alerts and routes are notified or lack of familiarity with the context) can be considered as a special vulnerable group. This way, our proposal is integrated into the definition of an Emergency Action Plan and complements the traditional processes by improving the way individuals equipped with smartphones receive and visualize alerts and evacuation routes during particular emergency situations.

In the rest of this paper we will first present the background of our work highlighting the shortcomings of existing technology and, after that identifying a set of criteria to accomplish in order to provide personalized emergency alerts and evacuation routes. In Section 4 we will describe the proposed solution and several use cases to clarify how our contribution could be applied to real emergency situations. Section 5 summarizes the results obtained from a usability and utility evaluation performed simulating the evacuation from one of the buildings of the Carlos III University of Madrid. Finally, Section 6 discusses some of the findings of this work where ontologies, mobile technologies and multimodal interfaces are combined to complement evacuation processes.

## 2. Background

As mentioned before, the preparedness phase has a crucial role in the emergency management process. In particular, the definition of an efficient EAP (Emergency Action Plan) could minimize the number of victims and damages. Within this scope, ICT represents a valid support for improving the efficiency and effectiveness of activities related to notifying and evacuating people in the affected area. For example, detection devices should be installed and a communication system must be provided to interact with the occupants of a building. Moreover, ICT can provide alternative channels and complementary to the traditional ones for, on one hand, providing a good warning mechanism for large and complex building or open space and, on the other hand, helping people to be aware of emergency situations and reach a safe place if required. In this way, detection of dangers and communication through alerts notifications and emergency evacuation systems can help ensuring a better reaction when an emergency occurs.

### 2.1. Alert Notification Systems

Some of the procedures in an EAP are related to notifying people that could be affected during an emergency. In this case, the role of ICT concerns the development of notification systems for communicating with the general public in emergency situations through different communication channels, like mobile phones, radio, television, cable networks, and wire services. Moreover, notifications can be transmitted using text descriptions as well as visual signals (e.g., strobe lights, rotating lights, and flashing lights) and audible signals (e.g., horns, bells, and sirens). Notified information ought to be appropriate to avoid panic and should state details of the emergency situation such as type, starting time, progress, affected area, and severity of disaster. Since notifications are critical in an emergency scenario, it is important to transmit them instantly to all the occupants in the affected area including emergency workers, such as the emergency manager, the evacuation chief and members of the Command Post.

In order to both collect and share information regarding notifications, a standard language is required, as posited in [10]. One of the technological challenges presented in [11] is the use of Standards for Notifications and Accessibility: “The key approach is to create standard message content with sufficient richness and meta-information to facilitate delivery in multiple modalities, either as an independent channel (voice/audio) or in synchronization with other forms (voice/audio and text)”. A standard format for emergency notifications is the OASIS Common Alerting Protocol (CAP) that has been widely adopted by several currently used systems. CAP standard supports textual presentations with recorded or synthesized speech presentation and multimedia content such as images. Table 1 presents a list of the most common alert notification systems with their available notification channels [12].

### 2.2. Emergency Evacuation Route

Concerning EAP procedures related to the evacuation process, an evacuation route is defined as a route that people must follow to escape from a dangerous area during an emergency situation. Focusing on indoor environments like workplaces, different types of emergency require the definition of different evacuation plans. For example, in case of a fire, people are required to go outside their workplace, but in a tornado it may be better to stay inside the building. There are three major aspects to consider when customizing emergency evacuation routes:

- Type of emergency: whether fire, explosion or earthquake, *etc.*
- Type of building: the building construction is very important to characterize the vulnerability due to the effects of disasters. Nearly every type of structure will be affected from major disasters; however, some buildings may collapse and others may be left with weakened floors and walls.
- Type of people: depending on the abilities (permanent or contextual) of affected people, some could need additional assistance.

Emergency evacuation routes are usually represented as maps generated from floor diagrams with signals that indicate the path to exits or safe places. Locations of exits, assembly points, and equipment such as fire extinguishers, first aid kits, and spill kits, should be included in the maps. Routes must be clearly marked, well lit, unobstructed and clear of debris at all times. Generally, an exit route consists of three parts: exit access, exit and exit discharge. An exit access is the portion of an exit route that leads to an exit, which is generally separated from other areas to provide a protected way of travel to the exit discharge. The exit discharge leads directly outside or to a street, walkway, refuge area, public way, or open space with access to the outside.

## 3. Criteria to be Achieved for Alerts Notification and Evacuation Routes ICT-Based Systems

Based on the requirements of the alert notification systems and emergency evacuation routes, we have defined a set of criteria that must be achieved by ICT-based systems, which can be integrated into the definition of an EAP in order to complement the traditional processes. We have derived this set of criteria from the study of current literature about this kind of systems [5–8] and from real systems that are used in emergency situations, including chemical, biological, radio-active, nuclear attacks and civil disturbances (see Table 1). It is worth noting that all criteria are related between them, but each one has a particular focus that contributes to reach the overall objective.

1. Accessibility for all
  The alert notifications and evacuation routes must be delivered to all kinds of people, including the more vulnerable, and in any context in order to promote equivalent life safety. On the one hand, people have different needs and skills and on the other hand, characteristics and circumstances of the emergency can affect the surrounding environment. Thus the system must support accessibility for all in every situation.
2. Personalized messages
  Emergency notifications must be personalized considering preferences expressed by users through the configuration service offered by the system. Such preferences concern data about the location of the user, capabilities of used device, visualization mode for evacuation routes and kinds of information to be alerted about. Whilst the previous criterion puts the stress on the need of creating accessible messages, this criteria focus on the capability of users to express their own preferences whether related or not with accessibility issues. In this way, contextual disability can be dynamically taken into account.
3. Multimodal alerts
  The use of different communication channels allows reaching users with diverse devices. Different communication channels (e.g., e-mail or SMS) must be available for reaching and notifying affected people during an emergency. Whilst the previous criterion focuses on the need to let users define their preferences, this one makes it explicit that the system has to support alternative communication channels whose availability might depend on the type of emergency.
4. Customized evacuation routes
  Evacuation routes vary with respect to the context of each person and the characteristics of the emergency situation. Thus, an evacuation route must be customized taking into account the user's location inside the building, personal circumstances (e.g., routes for wheelchair users usually ends in a meeting point), and characteristics of the situation (e.g., an unavailable corridor in the building).
5. Multimodal visualization
  Alert notification and evacuation routes must be presented in different ways in order to facilitate their understanding. For example, disabled and elderly people may not only need a map but also a speech-out presentation due to unclear sight or blindness, or contextually disabled people that might require indications of their current location.
6. Communication channel with the Command Post
  The Command Post of an emergency situation should know where the affected people are and how they are moving around the emergency area. Moreover, bidirectional communication channels between Command Post and affected people must be provided to maintain updated emergency situation in both senses (e.g., affected people can inform the Command Post about their injuries or about the description of a particular corridor).
7. Different alternatives for the services
  All provided services (e.g., user's location, network connection, *etc.*) must establish primary and alternative options to create redundancies, options in case of failures one fails during an emergency situation.
  Henceforth, we will address each of these criteria with a ‘#’ notation (e.g., #3 for criteria “multimodal alerts”).

### Analysis of the State-of-the-Art Systems

An analysis was made based on state-of-the-art solutions according to the criteria that we have defined (Table 2).

In particular, three alert notification systems have been analyzed: HUENS [5], CUCEM [6], and cAlert [7]. Furthermore, a complex emergency management system (The Digital Building [13]) and an indoor emergency evacuation service based on autonomous navigation system [14] are also discussed.

Although HUENS, CUCEM, and cAlert consider using multimodal alerts for notifying people of emergency through television and radio broadcast, email, telephone, text, and voice message, they do not make provision for impaired people. CUCEM system inspects only on notifying people of different emergencies without providing any personalized notification message based on user profile and other factors. Therefore, these three systems are not compliant with all defined criteria (#1-2, 4-6).

The Digital Building system sends alerts to users based on their location, emergency, and media; however, it does not consider needs and preferences of disabled and elderly people. Once in an emergency situation, the system distributes building information to laptop, smartphones, tablets and other web enabled devices. This information is based on user's location, environment, and kind of emergency. Nonetheless, user's abilities and impairments, levels of expertise, needs and preferences, and characteristics of used devices are not taken into consideration.

The indoor emergency evacuation service based on autonomous navigation system proposed by [14] provides user's location-based notification message. A textual view of evacuation routes with speech function is provided for disabled and elderly users. Also, the evacuation route is provided according to the device used (#5), and facilities in a building are included (for example, pictures showing the restroom for changing baby diapers). However, multimodal alerts (#3) are not used to meet needs of different kinds of users and devices. Moreover, mobile device calculates automatically the path to an exit with preinstalled building map in this system meaning that users are being lead to a predefined exit without taking into account the emergency situation context or the building current state. In fact, this system is not compliant with unforeseen situations like a specific exit is locked or broke down. Just as The Digital Building system, this proposal does not provide any communication service to contact the Command Post in case assistance or communication is required. Hence, both Digital Building and this system are not fully compliant with the criteria that we have defined.

## 4. A System Architecture for Managing Personalized Alerts and Evacuation Routes

Considering our previous analysis of existing systems, it seems none of the actual alert notification and evacuation route systems is completely compliant with the established criteria. Therefore, we propose a personalized alert notification and evacuation route system using portable terminals (e.g., mobile phones, PDAs, emails) in indoor environments based on the use of emergency plans. Our proposal uses existing hardware structures such as Wi-Fi access points and mobile phones. The system sends personalized alert notifications via multimodal alerts and provides multimodal visualization of adapted evacuation routes to all kinds of users. Alert notifications and evacuation routes are generated based on knowledge of each user's abilities and impairments, levels of expertise, needs and preferences, current location, available media and technology, the surrounding environment, and context of the emergency (e.g., severity and evolution). In particular, people that may need special assistance during an emergency situation are not only those that suffer from permanent disabilities but also other groups such as foreigners. Furthermore, people with contextual disability as a result of being unfamiliar with the building structure (e.g., a visitor) or with limitations from the event (e.g., fire smoke) are also involved in our target users.

The entire system is based on an extended version of an ontology called SEMA4A, already defined in [15]. The SEMA4A ontology has been developed to represent and relate main concepts in the domains of accessibility, technological infrastructures, emergency and evacuation procedures. Through SEMA4A, it is possible to infer information about the personalization of alerts and evacuation routes: for example, which kind of devices or communication infrastructures is most suitable depending on users' abilities. For the proposed mechanism, we use SEMA4A as basis for the personalization of both notifications and evacuation routes.

The mechanism makes use of mobile smartphones, increasing the probability of a successful evacuation of the affected people and making easier the evacuation process by informing users with updated information on where to go and how to reach the appropriate point. This system includes desktop applications for the definition of evacuation plans and for monitoring the affected people from the Command Post through the use of interactive maps. Moreover, it also includes a registration mechanism to save user profiles, which will be used during the emergency situation to personalize the notification alerts and evacuation routes. Finally, mobile smartphones will interact with the system sending its current location and receiving multimodal messages personalized according to the emergency situation, the personal context and the user profile.

For the evacuation, the proposed system selects the route that best meets the specified characteristics related to users and emergency situations among those defined in the official evacuation plan. In this way, delivered instructions do not interfere with procedures laid down by the Security Department in charge of their definition, but they represent a support to make the evacuation more efficient for different vulnerable groups that can still make use of smartphones.

**Figure 1.**
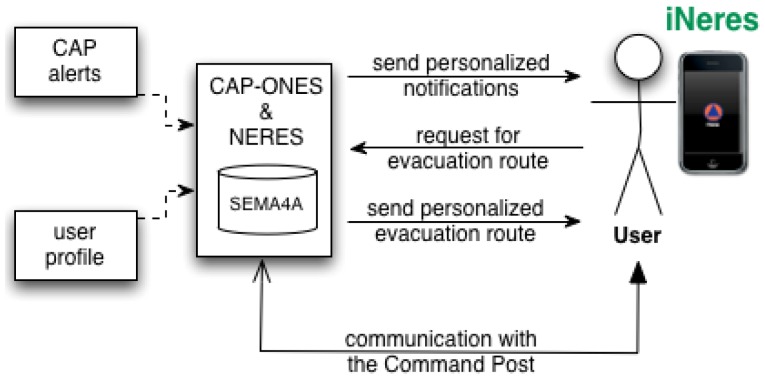
The system architecture for managing personalized alerts and evacuation routes.

Our proposal (Figure 1) is composed of three components:

- CAP-ONES (Common Alerting Protocol-based Open Notification System)
- NERES (Notification of Evacuation Routes in Emergency Situations)
- iNeres as the interface on the mobile client side

The next paragraphs describe these three components referring to the requirements met using their code.

### 4.1. CAP-ONES: Alert Notification

CAP-ONES is a server application that creates personalized alert notifications based on two inputs: an emergency alert in Common Alerting Protocol (CAP) format and a set of user profiles [10]. On one hand, the CAP message contains information about an emergency event including its status, description, severity, *etc.*, as well as a flexible set of additional information that can contain geographic data in different formats, support resources (video, images and/or text files) and system-specific parameters associated with the emergency alert. On the other hand, user profiles contain information about each user, including personal and contact data, user's abilities or impairments and possible devices. This set of user profiles is codified using a XML-based format, enabling interoperability for any system with XML support. CAP-ONES provides a Web interface to create user profiles where users can express their preferences (#2), but other systems can be implemented for enabling access through other devices. From these two inputs, the system extracts all the relevant information and performs queries on the SEMA4A ontology in order to infer potential ways to create and send the alert notification (#1). If there are any conflicts between preferences of users and data inferred from SEMA4A, the system gives priority to results obtained from SEMA4A because they consider additional variables related to the context (#1). The result set obtained from these queries provides with a set of available communication channels, devices and possible media that can be used to adapt the notification for the specific emergency event and each user profile, thus creating a personalized message (#1, #2) using the CAP alert data, and supporting multimodal alerts (#3, #5) depending on users abilities and devices capabilities. More details about the extraction of data and queries to the ontology are described in [15], and about the CAP-ONES application and the message personalization in [10].

### 4.2. NERES: Evacuation Plans

NERES is a server application for generating personalized evacuation routes based on user profiles and contextual information from an emergency. In order to obtain instructions for responding to the emergency, a client application sends a request to NERES that generates a personalized evacuation route according to the user's current location, user profile data, and the environmental situation of the emergency. This is achieved by two different mechanisms: an evacuation plan data model and a back-end server application. The server application employs the data model to generate and provide a personalized evacuation route for each affected person who is registered in the system.

**Figure 2.**
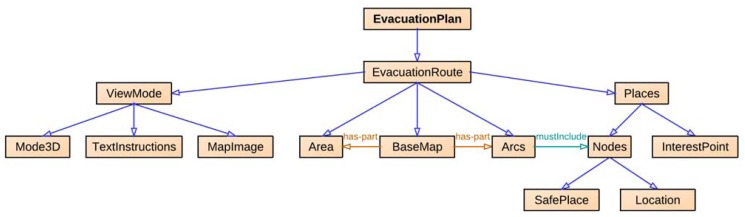
NERES: the evacuation plan data model.

The evacuation plan data model gathers the entities required to represent the subjects, objects and relationships involved in an evacuation process of an emergency plan (Figure 2). This model has a hierarchical structure with a root node, called *EvacuationRoute*, and five subclasses: *Places* as specific areas in the building; *Arcs* as paths between two nodes, an origin and a destination; *BaseMap* as the building structure; *Areas* as physical or conceptual sectors of the building; and *ViewMode* as the way the route can be presented to the user. Moreover, *Places* includes *InterestPoint* (e.g., location of fire extinguishers) and *Nodes* that consists of *Location* (e.g., offices) and *SafePlace* (e.g., emergency exits and meeting points). Through this model, an evacuation route (*EvacuationRoute*) is drawn over a map of the building (*BaseMap*) and it is defined as a linked set of arcs (*Arcs*), where the ending node (*Nodes*) is a safety place (*SafePlace*). Finally, each evacuation route can be visualized in three different ways (*ViewMode*): *MapImage*—an image of the building map, *Mode3D*—actual pictures of the building showing the way, and *TextInstructions*—text only instructions. In particular, the chosen mode for the visualization of a specific notification depends on characteristics of the user (e.g., her abilities and needs), the device (e.g., supported features as text-to-speech or image processing) and the context of use (e.g., limited vision due to the presence of smoke fire). For different users' profiles and contexts, the selected visualization mode could change. For example, a text description is adequate for f users with a visual impairment and a text-to-speech tool in their devices, while a map image is suitable for a person that knows the building and just wants to be sure about the position of exits or meeting points.

**Figure 3.**
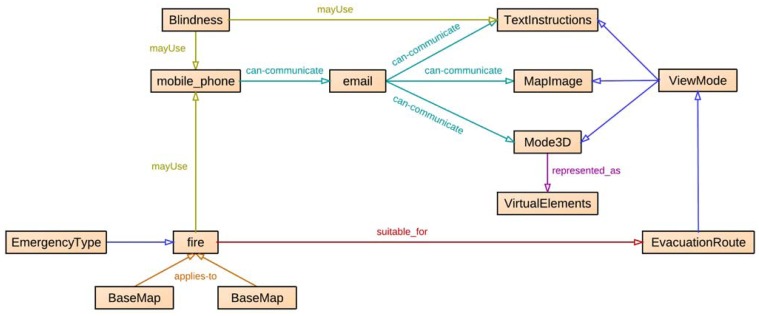
NERES: relationships of the evacuation plan data model with SEMA4A.

The evacuation plan data model has been added as additional knowledge in the SEMA4A ontology (Figure 3), defining relations between the evacuation route entities and existing entities. In this way, routes are adapted to the current event and personalized according to available media and user's abilities (#4, #5).

Each node from the route, including the ending node (*i.e.*, the safety place), contains a list of impairments that users with any of these impairments cannot access. Consequently, each arc also contains information about user impairments according to their corresponding nodes in order to verify the accessibility of the overall route. The final result is an evacuation route physically accessible to the user (#1).

Other relations have been defined with existing entities in SEMA4A regarding types of emergencies (e.g., relation *suitable_for* between *fire* and *EvacuationRoute* in Figure 3) and media (e.g., relation *can-communicate* between *mobile_phone*, *email* and subclasses of *ViewMode* in Figure 3). For instance, if the user is using a device that cannot display images, instructions should be presented in text-only mode.

**Figure 4.**
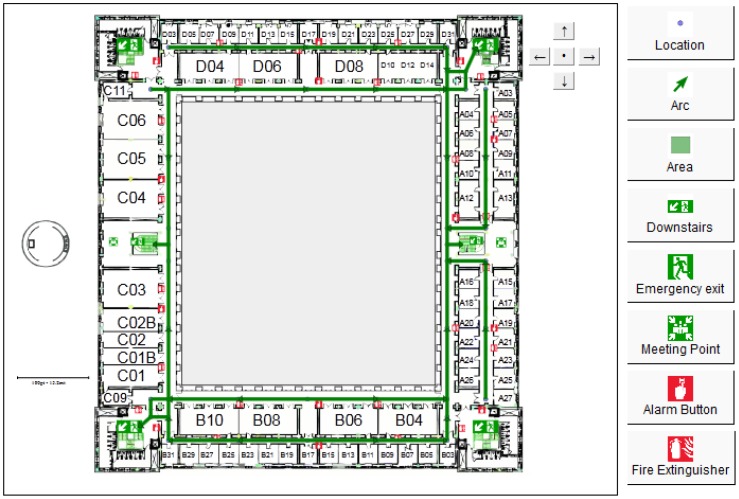
Definition of emergency evacuation routes in a particular floor.

NERES is executed in two different phases of the emergency management process: *preparedness* and *response*. During the *preparedness* phase, planners must define through NERES an evacuation plan for the building (Figure 4). In particular, they must define available evacuation routes for each floor, where origin nodes are all possible locations of users (*i.e.*, offices or rooms in a house) and destination nodes are predefined safety places. For each route, NERES verifies its accessibility by determining which user profile can access to the nodes and arcs that compose it. For example, from SEMA4A it is possible to infer that people with mobility impairment cannot go down the stairs, so NERES can identify an incompatibility between routes that use stairs and these specific profiles, storing these data for the *response* phase.

During the *response* phase, once users have received an alert from CAP-ONES, they can send a request to NERES to get the best evacuation route specifying their location (see Section 3.3 to see how it is calculated). On one hand, NERES takes into account contextual information about the current situation to establish if any area of the building has been affected by the emergency, in order to make unavailable evacuation routes inside such area. On the other hand, NERES considers user profiles and their location to determine the optimal route in the following way:

1. Using the location of the users, the system retrieves nearest nodes and arcs to determine available route to reach a safety point.
2. Considering contextual information about the emergency, the system filters out affected evacuation routes that are in dangerous areas.
3. Considering users' profile, the system determines which evacuation routes are incompatible with their abilities and impairments (e.g., no stairs for people with motor impairments).
4. With the final set of possible routes, the system chooses the shortest one as the most appropriate respect to users' abilities and emergency context.
5. Once the route has been selected, and in accordance with capabilities of devices, users' profile and emergency context, the evacuation route is notified with the most appropriate presentation mode.

NERES provides a personalized evacuation route taking into account the user and the emergency context using the SEMA4A ontology (#4). It includes three different modes to present both instructions and maps of evacuation routes (#5). The first one is the *Text-Only* view that displays a list of steps with a short description for each one. The second mode is the *3D* that shows an actual photo of the building for each step of the route with an arrow to indicate directions to follow. The last one is the *Map* that uses a map of the building to visualize the entire route, where each step is represented highlighting its related arc with a short description. The most appropriate visualization mode for notifying an evacuation route is determined considering users' profile, features of the device and emergency context. For instance, in case of a fire event in a building where the smoke could limit the vision, a graphic visualization mode (e.g., images of the environment) could help users in orientating themselves and reaching the exit. In the same situation, if the user has a visual impairment, she could not access to the graphic visualization mode and she should receive a text description for evacuation instructions to be read with a text-to-speech tool.

### 4.3. iNERES

iNERES is a mobile application that serves as interface for obtaining emergency evacuation routes, allowing to provide more contextual information and the possibility to communicate with the Command Post (#6). It exploits current technologies such as Wi-Fi fingerprinting, pattern recognition, Augmented Reality, chat service, and push notifications. Using this mobile application along with CAP-ONES and NERES, users can receive personalized alert notifications and be informed of constantly updated evacuation information and routes; thus, they can escape heading to the nearest safe exit or a safety place. In addition, with iNeres users are able to communicate with the Command Post, contributing to the evacuation process in avoiding further injuries or even death because of the delay in requesting for rescue.

The core concept is that user has a portable device to escape in an emergency situation. In an emergency situation, the user receives on her mobile device the alert generated from CAP-ONES based on the existing knowledge of the emergency context and the user profiles. After receiving the alert notification, iNeres allows getting a personalized emergency evacuation route by sending location data. The location data is elaborated in the back-end server to obtain user's location, and a set of evacuation routes leading to the exits nearest to the initial location is computed on the basis of data of the evacuation plan of the building, which is based on the evacuation plan data model as explained in Section 3.2.

A requirement of iNeres is to receive, through any channel, emergency alerts from server in order to be compliant with #3 and #7. This way, we try to optimize the probability of alerts received by users. In fact, different mobile communication mechanisms are adapted encompassing SMS, MMS, email, and push notifications (#3). We included also vibration in addition to audio alert for the accessibility of deaf people. Therefore, our proposal use alternative media to provide the alert service (#7), and the notifications are accessible to all kinds of users including those with special needs (#1).

Another relevant issue is the acquaintance of user's location in order to send user location-based evacuation route (#4). This can be achieved in four different ways:

1. Provided default location from user profile.
2. Fingerprinting mechanism based on Wi-Fi triangulation.
3. Pattern recognition mechanism to recognize the building tags.
4. Current location typed or selected by user.

**Figure 5.**
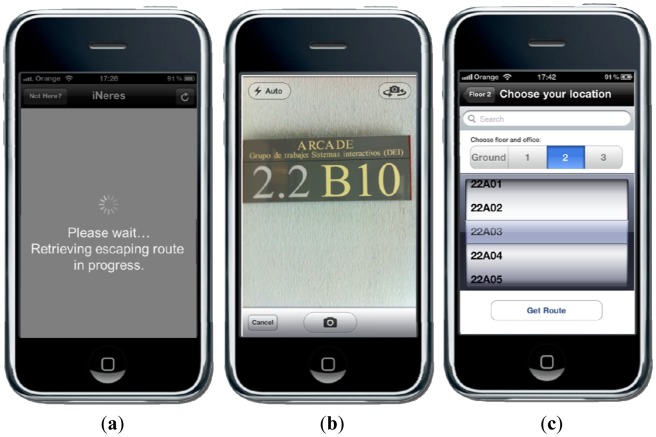
iNeres mechanisms for detection of user's current location: (**a**) Wi-Fi triangulation fingerprinting, (**b**) pattern recognition, and (**c**) user input.

For the last three cases (Figure 5), the iNeres application sends the user's location to the NERES server. As mentioned above, the location data can be text input by the user, a photo taken by the user for pattern recognition, and received signal strength (RSS) from all non-portable Wi-Fi access points, Bluetooth and GSM devices.

To simplify interaction, the default method used is the Wi-Fi fingerprinting mechanism (based on the application from [16]) that does not need direct interaction with user since the retrieving and forwarding of RSS are automatically running in the application background. This method creates fingerprints of each location by scanning available Wi-Fi networks and measuring the signal strengths for each one, storing this data altogether with a corresponding label. This way, when a user's location is needed, the application creates a fingerprint and requests for the location that better matches it. This approach follows a similar indoor localization system as [17]. In their contribution, Vera *et al.* have developed EDIPS (Easy to Deploy Indoor Positioning System) for allowing the indoor positioning of collaborators located in different offices based on Wi-Fi signal strength. Moreover, they have compared their solution with the current literature about indoor scenarios, obtaining a minimal effort for the initial setup with a high accuracy in positioning.

However, if the user has no Wi-Fi connection, we give the possibility to retrieve her location by using pattern recognition through user's 3G networks. An Optical Character Recognition (OCR) algorithm is used at the back-end server to compute the location once user takes a shot at the building room tag and sends it to the server. Here again, we try to adapt different channels to provide location-based evacuation route (#7).

The multimodal visualization of the evacuation route is made through the three different visualizations (#5) of the routes generated by NERES (*Text-Only*, *3D*, *Map*). Moreover, to exploit latest interaction paradigm Augmented Reality, a further presentation mode is added to iNeres, the a*ugmented reality* view that overlays information and direction to exit over the actual view obtained from mobile phone's camera view. Finally, it is worth noting that users can choose their preferred way of location detection and visualization mode from the application settings, but also during the walking through the notified route.

To improve the accuracy of evacuation routes in compliance with environment and user's current location, during escaping user can update information by clicking on an update button located on the top right corner of the user interface. Thus, the process of retrieving location data, sending to the server application and obtaining an evacuation route is repeated.

**Figure 6.**
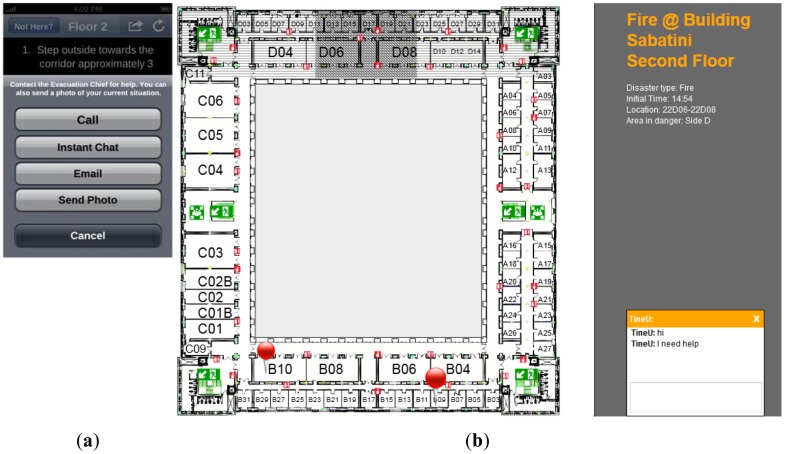
iNeres: communication with the Command Post. (**a**) iNeres tools to communicate with the Command Post. (**b**) Floor map includes information about emergency situation, affected people location and textual messages.

Furthermore, the communication between the Command Post and affected people during an emergency can help to provide relevant information to the affected people about how to behave during an evacuation process, as well as to indicate to the Command Post unexpected facts of the emergency situation. However, people do not save the contact of the Command Post, and they may call a global emergency number such as 911 or 112, but there still would be a delay time for redirecting the phone call to Command Post. Moreover, the use of smartphones can improve this communication with the use of multimedia content. For example, in case of an emergency where people are hard to reach due to debris or power went out, people could call or send a text message in order to receive instructions, Moreover, a user could send an image of the surroundings to the Command Post to facilitate getting rescued. Hence, our application includes a bidirectional communication channel (Figure 6) where users can report their situations to the Command Post (#6) via voice calling, text, chat, and email (#7), being able to send different formats of messages: audio, video, image, and text. The location of the user, if available, is sent along with any message to the Command Post. Figure 6 shows the interaction mechanism between a user and the evacuation chief. The (a) image depicts different modalities of communication on iNeres whereas the (b) image shows the front-end application for the Command Post. In the application for the Command Post, it is visualized an interactive map with the chat mechanism with people using iNeres and different data of interest, such as meeting points, exits, affected areas by the emergency (darkest square filled with diagonal lines in Figure 6(b)), unavailable areas (horizontal lines in Figure 6(b)), location of users subscribed to CAP-ONES (thumbtacks in Figure 6(b)). This information allows achieving a high level of situational awareness.

Finally, iNeres provides a configuration service to allow users changing the default preferences (#2). In particular, it is possible to indicate the location (Figure 5(c)), the favorite visualization mode for the evacuation route, the default language for the application and how to be alerted by push notifications.

In summary, our solution allows sending personalized alert notifications and evacuation routes on the basis of the knowledge contained in an ontology, which corresponds to the users' abilities, media and communication channels availability, and the emergency event characteristics and meets all the requirements stated in the previous section. Moreover, the mobile application allows attaching contextual information from the situation, in particular the user's location data for a better adaptation and personalization of the notification. Additionally, four visualization modes for supporting accessibility of the evacuation directions are supplied. Finally, a mechanism for bidirectional communication with the Command Post is developed.

### 4.4. Use Case

**Figure 7.**
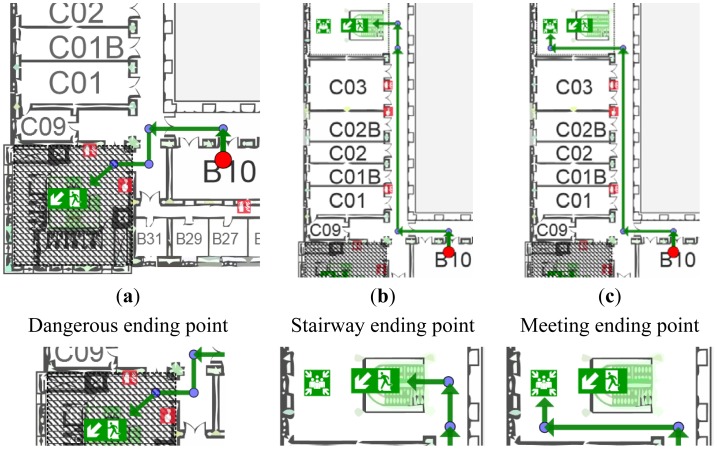
Potential evacuation routes according to user's location: (**a**) dangerous area, (**b**) stairway ending point, and (**c**) selected route.

Let us consider an example: a user with limited mobility is subscribed to the system using a personal device with image display capabilities. The user receives from CAP-ONES an alert notification about a fire emergency in the building where she is and an evacuation procedure has started to avoid personal damages. She launches iNeres application that calculates her location (e.g., office number “2.2.C01B”) and sends a request to NERES for obtaining a personalized evacuation route according to her profile. Considering her location, NERES identifies three potential routes with different ending points, as shown in Figure 7.

NERES verifies each route according to contextual information of the emergency and detects that route (a) passes through an unavailable area due to the current emergency situation (square filled with diagonal lines in Figure 7(a)), therefore the route is discarded. After that, the system analyzes the second route (Figure 7(b)), which includes a stairway as ending location, for this reason it is also discarded because the user has a motor impairment and she cannot go downstairs without an appropriate support. Last route (Figure 7(c)) is detected as the most appropriate one, having a meeting point as ending node where the user should receive assistance for escaping the building. If there were other alternative routes accessible for the user, the application would compare the sum of the distances of each route's arcs and select the one with the minimum distance between origin and destination.

**Figure 8.**
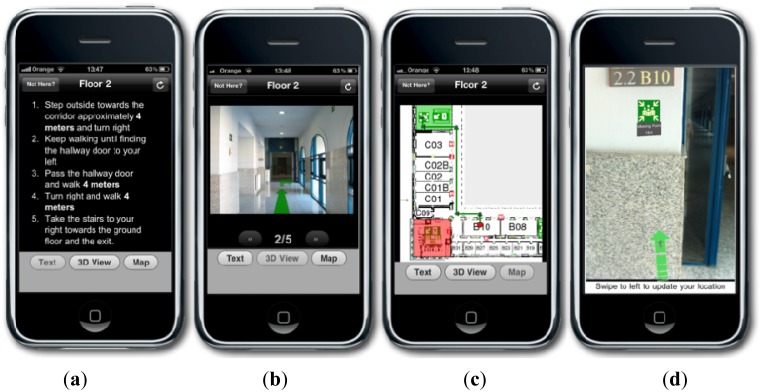
iNeres: Multimodal visualization of the evacuation routes. (**a**) Text-only view. (**b**) 3D visualization. (**c**) Map visualization. (**d**) Augmented Reality view.

Finally, the system considers both abilities of user's profile and capabilities of used device in order to select an appropriate visualization mode: in this example, the user knows the building and the *Map* mode could help her in finding quickly where she has to go to receive assistance. At this point, NERES sends the personalized route and selected visualization mode to iNeres that redirects these data to the user in response to her needs. The user receives the route with the *Map* view (Figure 8(c)) and the possibility to choose another mode (Figure 8(a,b,d)).

## 5. The Evaluation of iNeres

In order to evaluate the utility of the mechanism and the usability of iNeres, we have performed a user study in a real setting. During a simulated fire alarm, users have been asked to evacuate one of the university buildings using iNeres for receiving notifications, visualizing the proper route and communicating with the Command Post.

### 5.1. Involved Participants

The evaluation has been performed with twelve students (ten males, two females) of the University Carlos III of Madrid, both in Master and Ph.D. programs, all of them between 18 and 35 years old. This group of individuals has been selected for their contextual disability related to their low familiarity with the structure of the building where the test has been carried out. In particular, they do not have information about where exits or meeting points are located and, in case of an evacuation, this lack of information could make them feel disoriented. For this reason, people with this kind of profile can be considered as contextually disable and, therefore, a vulnerable group with special needs.

Moreover, selected evaluators were grouped into two different categories depending on their experience with mobile operating systems and common mobile user interfaces: 33% belongs to *low expertise* group, 67% belongs to *high expertise* group. Finally, they were asked about previous experience with systems similar to iNeres, resulting that just one of them had used an emergency management system for earthquake alarms.

### 5.2. User Study

The overall duration of the user study for each evaluator was around 30 minutes, consisting of three different phases: *training*, *task execution* and *questionnaire*. During the *training* phase, a brief explanation of 10 minutes was given in order to clarify the purpose of the experiment and to introduce the iNeres application, its functionalities and, in particular, how the communication with Command Post works through the chat service.

Successively, involved users have participated at the *task execution* phase, simulating a fire evacuation process supported by iNeres for navigating the route and reaching the exit. While the emotional state of people affected by an emergency and, consequently, an evacuation procedure is a very interesting aspect to take into account, in case of a simulated emergency event it is not possible to retrieve data about how the stress or the fear could influence the evaluation.

**Figure 9.**
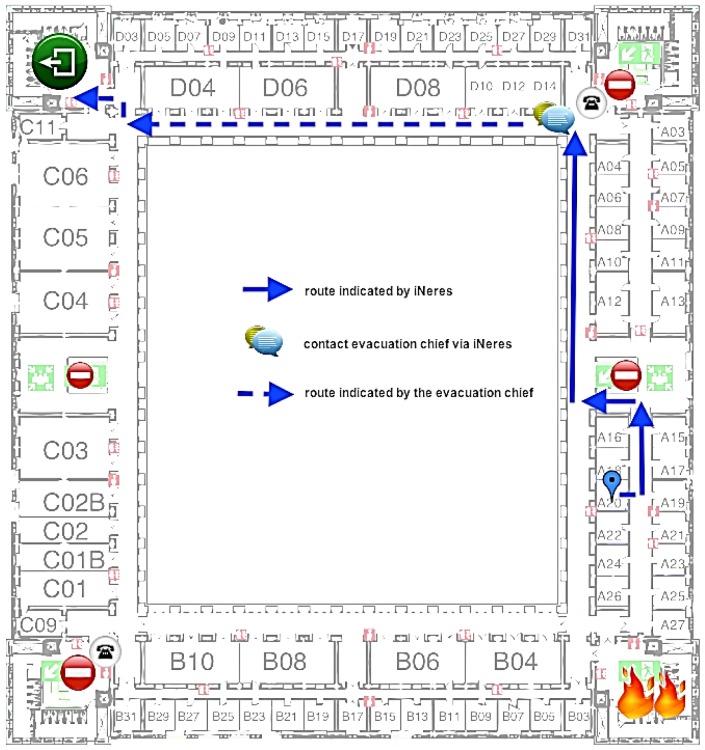
Map with the evacuation route provided by iNeres starting from the user's location and the point where user has contacted with the Command Post.

The proposed scenario describes the simulation of a real case in a building of Carlos III University of Madrid: an occasional visitor is participating at a meeting in one of the offices of the second floor when a fire started in a chemistry laboratory of the first floor. At the same time, the user receives an alert notification on her mobile phone alerting about the occurred emergency and suggesting the use of iNeres to reach the nearest exit. The user is able to explore all available functionalities, including the multimodal visualization and the chat service to contact the Command Post if she is in trouble. The map with the evacuation route provided by iNeres starting from the user's location is showed in Figure 9.

Once the *task execution* phase is completed, each evaluator was asked to fill a usability questionnaire. The questionnaire was designed for collecting opinions and suggestions about the experience, following the guidelines proposed in [18]. It is composed of four groups of questions: *system and past experiences* (from Q1 to Q3), *system acceptance* (from Q4 to Q8), *task execution* (from Q9 to Q10), and *user satisfaction* (form Q11 to Q16).

**Figure 10.**
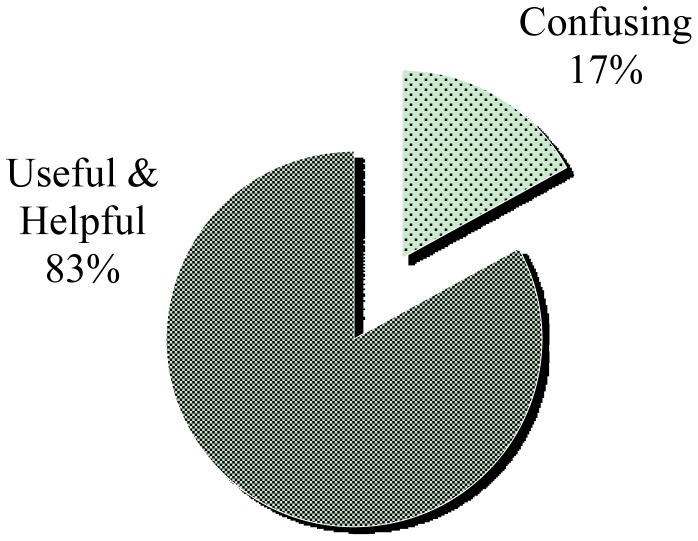
Graphical visualization of results of first part of the questionnaire (Q3).

From the first group of questions (Q1 to Q3), we gathered information about user profiles and a general comment about iNeres. Analyzing and categorizing collected opinions, we can conclude that the 83% of participants in the study have found very useful and helpful a mobile application for visualizing and navigating evacuation routes (Figure 10). Moreover, we received positive feedbacks about the simplicity and easiness of the interface, as suggested in the following statements collected from involved participants:

“It is a very useful application for visualizing evacuation routes.”

“Simple interface, few steps required to operate. Nice!”

“The interface was good, clear and simple. Positioning was reasonably accurate.”

“In general, it's a versatile and useful system, i.e., well-though different supporting interfaces.”

However, the 17% of users have found that different visualization modes could potentially confuse them during a crisis situation:

“I liked to have a ‘compass’ in my hands to help me get out, but I got a little confused by the different visualization modes and kept switching between them (panic! :-)”

“Choice of evacuation views can potentially confuse novice users.”

From the second group of questions (from Q4 to Q8), we obtained information about the system acceptance. In particular, we evaluated the user experience asking for five statements (as summarized in Table 3) over a Likert scale from 1 (strongly agree) to 5 (strongly disagree). In this case, we have followed the methodology suggested in [19].

In general, users agreed about the helpfulness of the training phase and the good understanding of provided information, as confirmed by computed means and standard deviations, except for the Q8 about the performance of iNeres. Concerning last question (Q8), obtained results match with several problems we noticed during the *task execution* phase. In fact, there are four Wi-Fi access points in the floor where the test was carried out and smartphones have to change from one to another in order to remain connected. This change affects the performance of the system since it requires several seconds to reconnect, affecting the user satisfaction. For this reason, evaluators have found iNeres quite slow for navigating visualized routes and for communicating with the Command Post.

Despite performance problems, users have achieved a good level of satisfaction completing the task, as shown in Table 4. The satisfactory experience is due to the interface and the simple interaction for accessing to the main functionalities, as well as the amount of time for completing the evacuation.

**Figure 11.**
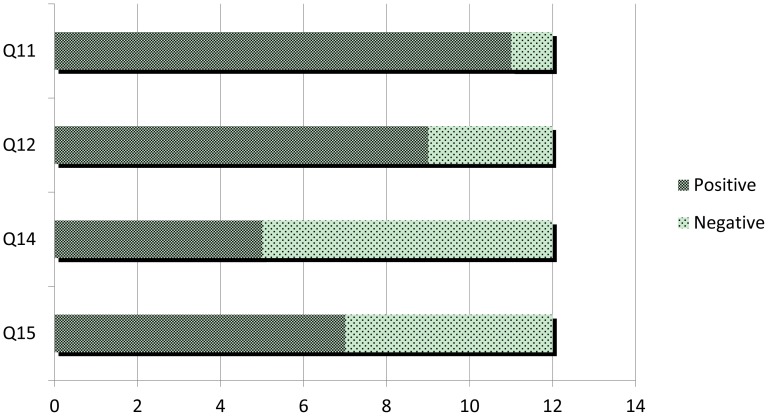
Graph visualization for results of the 4th part of the questionnaire (Q11, Q12, Q14, Q15).

In the last part of the questionnaire, we have collected data about the user experience with iNeres. First of all, we have analyzed the usefulness of a mobile application as support for evacuation. The first conclusion we drew out from these data is that when an emergency occurs and an evacuation process begins, almost all participants preferred to bring their own mobile phones with them (Q11, Figure 11). Strictly related to this, iNeres has been considered a more useful support for escaping in contrast to looking for static signals on walls and doors (Q12, Figure 11). After that, we have asked for the innovation of provided services, obtaining a high agreement within an overall consensus (Q13, Table 5). As already analyzed in the second part of the questionnaire, participants encountered several problems both for visualizing routes and contacting with the Command Post (Q14 and Q15, Figure 11).

Finally, evaluators had the opportunity to suggest further services, features or improvements in the last question they would like to include in the system. The proposals were:

- Concerning the communication with the Command Post, while the email feature seems to be useless, the voice call could be more useful compared to the chat mechanism.
- It could be helpful to provide the chat with a set of default or well-known expressions to avoid writing the whole message in order to save time, as suggested by one of the evaluators in the following statement:
  “In fact, whenever you contact the chief (EC), it only means that you are stuck. Thus simplifying this task would be very beneficial.”
- An improvement for the pattern recognition mechanism for indoor localization of user's current position could be the employment of a QR code as backup for the Internet connection in predefined points where there is a low coverage.
- In order to support the cooperation with other people, it could be interesting to study the possibility of visualizing the position of others using the system in the vicinity.
- For the evacuation route map visualization, a suggestion is to enable zoom features on the map.

Summarizing the results obtained from the user study, we can conclude that users have found the application easy to understand, to learn and to use. In particular, they have appreciated the usage of concise terminology and messages with a clear understanding of their meanings. Moreover, interactions with provided functionalities were simple and rapid thanks to employing a minimum number of steps to complete tasks. Finally, we have also collected negative opinions mainly related to performance problems, such as Internet connection availability and too many visualization modes for evacuation routes. In fact, during an emergency, users feel stressed and worried about their own situation and they prefer direct instructions (*i.e.*, succinct terminology and messages) rather than multiple options to choose (*i.e.*, different visualization modes).

## 6. Conclusions and Future Work

In the literature there are several contributions in the area of evacuation and accessibility: models and simulations have been proposed to improve the efficiency of the evacuation process, studying mainly trajectories of individuals or groups of individuals and how people with disability could affect them. Analyzing these contributions and existing notification and evacuation systems, we have identified a set of seven criteria for making ICT-based systems an efficient support for traditional evacuation procedures. These criteria guarantee that all involved individuals in an emergency situation are provided with the more adequate information in the more effective way, as stated in the first (*Accessibility for all*) and the second (*Personalized messages*) criteria, and more specifically in the others (*Multimodal alerts*, *Customized evacuation routes*, *Multimodal visualization*, *Communication channel with the Command Post*, and *Different alternatives for the services*). Moreover, these criteria are the basis for defining a personalization service that takes into account not only abilities of involved users, as required by the Emergency Action Plan in working places, but also factors related to available technologies (*i.e.*, communication channels and visualization modes) and contextual information about the emergency and the surrounding environment.

In order to prove the applicability of proposed criteria, in this paper we have also introduced a mechanism for generating personalized alert notifications and evacuation routes based on them. The aim of the proposed mechanism is to improve the escape process for, in particular, vulnerable groups and individuals that should need a special attention, notifying them with information about appropriate routes and procedures to follow. Other similar proposals doesn't take into account the personalization and the accessibility criteria, not allowing the sending of personalized and accessible messages that make easier the escape of individuals. The use of an ontology, SEMA4A, allows the creation of adaptable information according to the current situation and the particular needs of the individual, both to the notification of alerts (through CAP-ONES) as in the definition of evacuation routes (through NERES). Our proposal adapts the information of the Emergency Action Plan, complementing the traditional processes of evacuation and improving the way individuals equipped with smartphones (including the iNeres app) receive and visualize alerts and evacuation routes during particular emergency situations. Our proposal wants to maintain informed and to help to isolated individuals since the lack of information in an emergency situation can provoke misunderstandings. This solution is not so effective when the evacuation involves a group of people who are interacting among them since a number of additional factors, like the shared common knowledge, are used by them in order to decide what do in any moment.

The usability and understandability of proposed interface in iNeres has been assessed through a user study performed in a fire simulation in an indoor environment. This evaluation included not only finding any usage problems, but also collecting opinions and suggestions from participants. In general, positive feedbacks were received in particular about the easiness to understand, to learn and to use of iNeres. Although the evaluation was made in a controlled environment, similar results are expected in a real fire since evaluators were required to find the exit in the shorter time. Moreover, due to the relevant role of mobile phones on our daily life, users found very innovative the idea to use them as support for escaping instead of static signals on walls and doors. This kind of devices is not full spread into the population and others no so sophisticated are usually used, for example by elder people, so our mechanism must be complemented with others or the emergency plan could force to carry a compatible device with our proposal to everybody, employee and visitors. Several location mechanisms in iNeres are provided to give alternatives to get the current localization, but different performance problems were detected, mainly related to the establishment of Internet connection through different Wi-Fi access points. Finally, the proposed mechanism could be applied both for planning and response activities, taking advantages from the personalization and defining any common tasks.

Building upon the work we have presented in this paper, future contributions will be oriented towards improving the augmented reality visualization of emergency evacuation routes by adapting pattern recognition techniques to recognize objects and places, such as for example coffee machines or plants. In this way, we will provide our mechanism with an accurate acquisition of users' positions and overlay step-by-step evacuation instructions over reality. Moreover, we could use images or videos from monitoring systems available in the considered environment for positioning or managing the entire process. In addition, we will take into account problems occurred during the user study and collected suggestions from user experiences, in particular concerning localization with Wi-Fi triangulation and spent time for the entire evacuation process. Another interesting idea for future contributions is to analyze the impact of using iNeres during a group evacuation. While in the performed case study considered for the evaluation we have presented here each participant evacuated the building individually, we could image a different scenario where a group of people with special needs and abilities have to be evacuated. Moreover, we could spread the usage of bidirectional communication channels for supporting isolated people in being aware about their own situation.

## Figures and Tables

**Table 1.** List of alert notification systems (Adapted from [12]).

**System**	**Webpage**	**Notification**
3n	http://www.3nonline.com/	Phone, E-mail, Pager, Fax, SMS, PDA
AlertFind	http://www.messageone.com/crisis-communications/	Phone, E-mail, Pager, Fax, SMS, PDA
Arce	https://arce.dei.inf.uc3m.es/	Web pages, E-mail
Command Caller	http://www.voicetech.com/	Phone, E-mail, Pager, Fax, SMS, PDA
RapidReach	http://www.rapidreach.com/	Phone, Pager, Fax, SMS y E-mail
Sahana	http://www.sahana.lk/	Web pages
Sigame	http://www.sigame.es/	Web pages
SWN	http://www.sendwordnow.com/	Phone, E-mail, Pagers, SMS, MMS, VoIP, Skype, Chat y PDAs
WAVES Alerter	http://www.madah.com/products/	Phone, E-mail, Fax, PDA, TDD/TTY

**Table 2.** Analysis of the State-Of-The-Art Systems with our Defined Set of Criteria.

**Criteria**	**HUENS**	**CUCEM**	**cAlert**	**The Digital Building**	**System of Inoue *et al.***
#1	no	no	no	no	no
#2	yes	no	yes	yes	yes
#3	yes	yes	yes	no	no
#4	no	no	no	no	no
#5	no	no	no	yes	yes
#6	no	no	no	no	no
#7	yes	yes	yes	no	no

**Table 3.** Statements, mean and standard deviation for the second part of the questionnaire.

**ID**	**Questions about System Acceptance**	**Mean**	**Standard Deviation**
**Q4**	The system was easy to learn (positive)	2.33	1.07
**Q5**	The system was easy to use (positive)	2.5	1.24
**Q6**	The terminology and messages used by iNeres were understandable (positive)	1.58	0.79
**Q7**	The organization of information presented by iNeres was clear (positive)	2.58	1.24
**Q8**	The system was slow in some stages of usage (inverted)	2.33	1.23

**Table 4.** Statements, mean and standard deviation for the third part of the questionnaire.

**ID**	**Questions about Task**	**Mean**	**Standard Deviation**
**Q9**	Do you think that it is easy to complete this task? (positive)	2.08	0.9
**Q10**	Do you think that the amount of time it took to complete this task is adequate? (positive)	2.33	1.3

**Table 5.** Statements and results for the fourth part of the questionnaire (Q13).

**ID**	**Questions about User Reaction**	**Mean**	**Standard Deviation**
**Q13**	Do you think that the services offered by this system are innovative and that most people would find the system useful?	1.83	1.03

## References

[1] Perry R.W., Lindell M.K. (2003). Preparedness for emergency response: Guidelines for the emergency planning process. Disasters.

[2] Turoff M., Hiltz S.R., White C., Plotnick L., Hendela A., Yao X. (2009). The past as the future of emergency preparedness and management. Int. J. Inf. Syst. Crisis Response Manag..

[3] US Department of Labor Evacuation Plans and Procedures eTool: What is an Emergency Action Plan?. http://www.osha.gov/SLTC/etools/evacuation/eap.html.

[4] Van de Walle B., Turoff M. (2007). Emergency response information systems: Emerging trends and technologies. Commun. ACM.

[5] Harvard University Emergency Notification System (HUENS) http://www.uos.harvard.edu/opscenter/emergency_management.shtml.

[6] Campus Emergency Management Clemson University (CUCEM) http://www.clemson.edu/campus-life/campus-services/cufd/campusemergencymanagement/index.html.

[7] cAlert: The University of Chicago https://calert.uchicago.edu/.

[8] Koo J., Kim Y.S., Kim B. (2012). Estimating the impact of residents with disabilities on the evacuation in a high-rise building: A simulation study. Simulat. Model. Pract. Theory.

[9] Manley M., Kim Y.S. Exitus: Agent-Based Evacuation Simulation for Individuals with Disabilities in a Densely Populated Sports Arena.

[10] Malizia A., Acuña P., Onorati T., Diaz P., Aedo I. (2009). CAP-ONES: An emergency notification system for all. Int. J. Emerg. Manag..

[11] Sullivan H.T., Häkkinen M.T., Piechocinski D. (2009). Improving participation, accessibility and compliance for campus-wide mobile emergency alerting systems. Lect. Notes Comput. Sci..

[12] Malizia A., Onorati T., Diaz P., Aedo I., Astorga-Paliza F. (2010). SEMA4A: An ontology for emergency notification systems accessibility. Expert Syst. Appl..

[13] Emergency Management: Archaio, The Digital Building http://archaio.com.concentric.com/emsolution.html.

[14] Inoue Y., Sashima A., Ikeda T., Kurumatani K. Indoor Emergency Evacuation Service on Autonomous Navigation System Using Mobile Phone.

[15] Malizia A., Onorati T., Bellucci A., Diaz P., Aedo I. Interactive Accessible Notifications for Emergency Notification Systems.

[16] Bolliger P. Redpin—Adaptive, Zero-Configuration Indoor Localization through User Collaboration.

[17] Vera R., Ochoa S.F., Aldunate R.G. (2011). EDIPS: An Easy to Deploy Indoor Positioning System to support loosely coupled mobile work. Pers. Ubiquit. Comp..

[18] Preece J., Rogers Y., Sharp H. (2002). Interaction Design: Beyond Human-Computer Interaction.

[19] Dix A., Finlay J., Adowd G., Beale R. (1998). Human-Computer Interaction.

